# 3D-Printed Patient-Specific Instrumentation Technique Vs. Conventional Technique in Medial Open Wedge High Tibial Osteotomy: A Prospective Comparative Study

**DOI:** 10.1155/2020/1923172

**Published:** 2020-11-15

**Authors:** Yunhe Mao, Yang Xiong, Qi Li, Gang Chen, Weili Fu, Xin Tang, Luxi Yang, Jian Li

**Affiliations:** ^1^Department of Sports Medicine, West China Hospital, Sichuan University, No. 37, Guoxue Alley, Chengdu, China; ^2^Sichuan International Expo Group, Chengdu, China

## Abstract

**Purpose:**

The purpose of this study was to compare the accuracy and clinical outcomes of the medial open wedge high tibial osteotomy (MOWHTO) using a three-dimensional (3D-) printed patient-specific instrumentation (PSI) with that of conventional surgical techniques.

**Methods:**

A prospective comparative study which included 18 patients who underwent MOWHTO using 3D-printed PSI technique (3D-printed group) and 19 patients with conventional technique was conducted from Jan 2019 to Dec 2019. After the preoperative planning, 3D-printed PSI (cutting guide model) was used in MOWHTO for 3D-printed group, while freehand osteotomies were adopted in the conventional group. The accuracy of MOWHTO for each method was compared using the radiological index obtained preoperatively and postoperatively, including mechanical femorotibial angle (mFTA) and medial mechanical proximal tibial angle (mMPTA), and correction error. Regular clinical outcomes were also compared between the 2 groups.

**Results:**

The correction errors in the 3D-printed group were significantly lower than the conventional group (mFTA, 0.2° ± 0.6° vs. 1.2° ± 1.4°, *P* = 0.004) (mMPTA, 0.1° ± 0.4° vs. 2.2° ± 1.8°, *P* < 0.00001). There was a significantly shorter duration (*P* < 0.00001) and lower radiation exposures (*P* < 0.00001) for the osteotomy procedure in the 3D-printed group than in the conventional group. There were significantly higher subjective IKDC scores (*P* = 0.009) and Lysholm scores (*P* = 0.03) in the 3D-printed group at the 3-month follow-up, but not significantly different at other time points. Fewer complications occurred in the 3D-printed group.

**Conclusions:**

With the assistance of the 3D-printed patient-specific cutting guide model, a safe and feasible MOWHTO can be conducted with superior accuracy than the conventional technique.

## 1. Introduction

Medial open wedge high tibial osteotomy (MOWHTO) is a well-established surgical procedure in dealing with early or mild stage of knee osteoarthritis (OA), and this native knee-preserving surgery could ensure long-lasting clinical success (>10 years) in the overall treatments of knee OA [1, 2]. MOWHTO is typically applied for the correction of varus malalignment of the lower extremities in isolated medial compartment arthritis of the knee [3–5]. If accurately performed, MOWHTO has the potential to delay or even possibly prevent the development of end-stage OA, by shifting the weight-bearing axis toward the lateral compartment [3, 6]; the loading is redistributed, and knee function is thereby restored and could avert total knee arthroplasty (TKA).

Nevertheless, the downsides of this procedure remain notable. Except for the high rates of knotty local complications, including increased tibial slope, hinge fractures, infections, and delayed union [7, 8], the main obstacle lies in the accuracy of performing osteotomy [9]. A successful MOWHTO requires the angular correction to be achieved accurately in both the sagittal and coronal planes, making it fairly challenging to determine the accurate osteotomy opening distance with the current conventional techniques [4, 10]. The systematic review by Van den Bempt et al. [4] revealed that the accuracy of conventional MOWHTO was below 75% in 8 out of 14 cohorts. Small errors in osteotomy positioning can lead to severe local complications such as lateral cortex fractures [11], and minor inaccuracy of angular correction in the coronal plane hinders the long-term success of this operation and even accelerates the progression of OA [12]. For the small tolerance for errors and the complexity for mastery, conventional MOWHTO gradually comes to be an unfavorable alternative [13].

However, the newly developed ancillary technology in the modality of 3D-printed patient-specific instrumentation (PSI) may be a solution to the accuracy requirements of HTO planning and execution [13]. This technique was initially carried out in maxillofacial surgery [14]; however, its practicability was more adequately embodied in the later orthopedic studies [5, 15–17]. The feasibility and proof-of-concept study by Victor and Premanathan [17] reported PSI for 14 cases of osteotomy around the knee yielded satisfactory outcomes, suggesting it to be a prospective solution. In the study by Van Genechten et al. [5], similar competent postoperative overall results were achieved by MOWHTO with the assistance of the 3D-printed PSI. Moreover, with a safer and faster osteotomy, it allows orthopedists to perform more concomitant surgeries at one time, such as meniscectomy and anterior cruciate ligament reconstruction (ACLR) [18–20]. Nevertheless, despite all these desirable superiorities, there was an evident scarcity of prospective comparative studies with robust evidences to prove the clinical advantages of PSI over conventional techniques in MOWHTO.

This study is thus designed to identify the safety, feasibility, and reliability of 3D-printed PSI for MOWHTO and to determine whether this novel technique could achieve better clinical outcomes and accuracy, when compared with conventional MOWHTO, in terms of correcting the varus malalignments in patients with isolated medial compartment OA. The null hypothesis was that MOWHTO with PSI technique could offer better clinical outcomes, fewer complications, and more accurate realignment over the traditional MOWHTO.

## 2. Methods

### 2.1. Patients

18 MOWHTO surgeries with 3D-printed PSI technique and 19 conventional MOWHTO were conducted between Jan 2019 and Dec 2019 at Sports Medicine Center, Western China Hospital, Sichuan University. The study was approved by the Health Sciences Research Ethics Board at Sichuan University and at the local research ethics board at each institution (ID: 2018534)

Patients were considered for inclusion if they meet the following criteria: (1) age between 35 and 60 years old; (2) isolated medial compartment OA, Kellgren-Lawrence grade ≤ III; (3) radiological evidences for varus malalignment (varus > 6°, mechanical medial proximal tibial angle, mMPTA < 85°); (4) ROM: flexion ≥ 120°, lossofextension ≤ 10°; and (5) outer bridge grade for cartilage injury < IV (defect area < 2.5cm^2^). Patients were thoroughly informed about the pre- and postoperative radiology protocol, the planning procedure, and the PSI surgical technique. On a voluntary basis, for the patients who agreed to take HTO at our medical center, either with novel PSI or conventional technique, preoperative hip-to-ankle double-limb weight-bearing X-ray view of the knee (anteroposterior (AP), lateral view), whole lower limb CT scan of both sides, and MRI of the affected knee were taken. The same imaging protocol was repeated 3 months and 12 months after surgery to evaluate the angular correction in both sagittal and coronal planes, the accuracy of hardware positioning, the condition of the cartilage, and the healing of the osteotomy.

All included patients in both groups had completed the prementioned radiology protocol and clinical assessments. The demographic characteristics of the included patients were shown in Table 1.

### 2.2. Preoperative Planning

**Figure 1:**
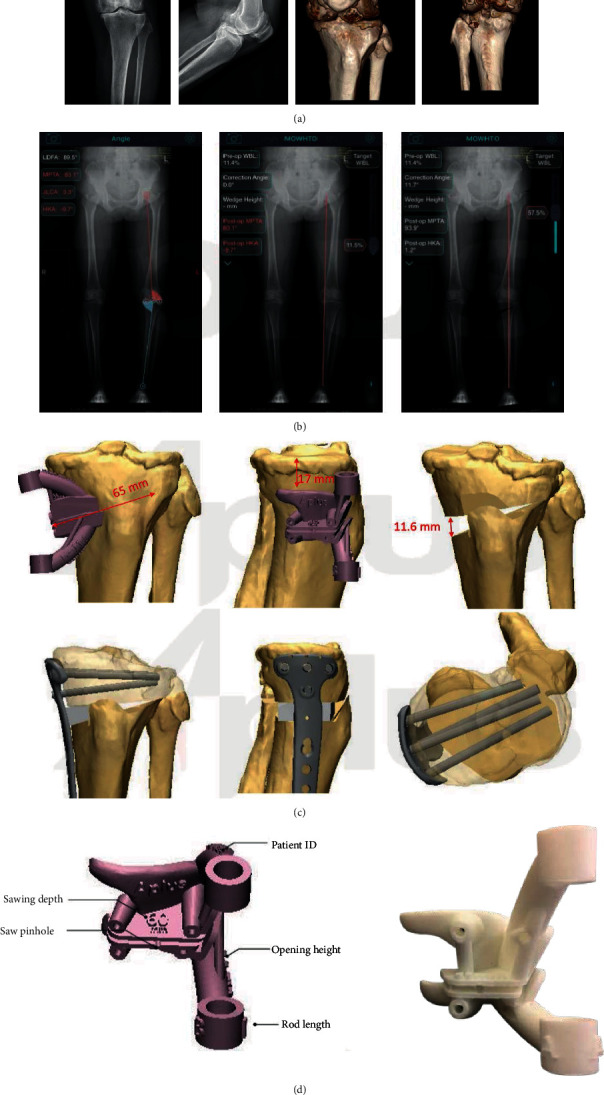
Female, 43 ys, suffered from left knee varus deformity, osteoarthritis (medial compartment, K-L III), and synovial chondromatosis (a). Preoperatively planed optimal mFTA and mMPTA were measured (b), osteotomy was simulated (c), and PSI was printed (d).

With reference to the methodology and parameters provided by Chieh-Szu et al. [21, 22], under the guidance of a radiology engineer (B.J.), by using the DICOM (digital imaging and communication in medicine) data, continuum-based tibial and fibular models from the CT image (slice thickness: 1.5 mm; image resolution: 512 × 512 pixels) were reconstructed as the intact model. A computerized osteotomy simulation software (OsteoMaster) was adopted to create the 3D bone anatomy virtual models of the lower limbs (Figure 1).

After the optimal sagittal and coronal correction angles, depth, width, height, slope, and position of the osteotomy were determined, the PSI cutting guide model was then built accordingly using additive layer manufacturing (3D printing) for the accurate osteotomy in the material of hydroxyapatite. Every osteotomy case was planned by a single investigator (Y.X.) who was highly trained in working with 3D medical software programs according to the protocol previously mentioned (Figure 1).

### 2.3. Surgical Procedures

Surgeries were performed by a single senior surgeon (J.L.). Firstly, intra-articular procedures were performed, arthroscopy was taken at each patient in the exploration for concomitant diseases, and articular debridement, free body removal, meniscectomy, or ACLR were conducted if necessary.

**Figure 2:**
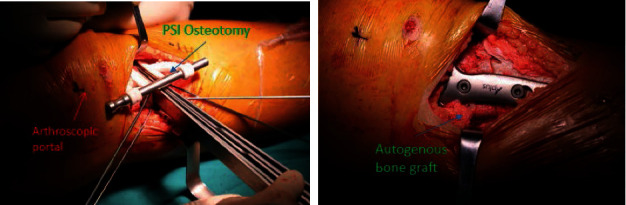
In operation, firstly, arthroscopic debridement of the synovial chondromatosis was conducted. Then, a two-planar osteotomy was performed, the wedge shape gap was widened and fixed at the predetermined angle via a metal bar stabilizer, and the locking plate was tightly fixed by screws. Autogenous bone grafting was implanted.

For the PSI technique, a 10-cm vertical medial tibia skin incision was made 2 cm below the tibial articular surface; then, the pes anserinus tendon was explored and loosen to allow greater surgical exposure; the tibial insertion of the superficial layer of the fibular collateral ligament (FCL) was then released, and osseous landmarks were made for the PSI cutting guide model positioning, fixed by saw pins. Then, the two-planar osteotomy was performed by a swing saw through the cutting grooves of the guide model, the wedge shape gap was widened length by length with steel rulers and fixed at the predetermined angle via a metal bar stabilizer, then a distractor was used to maintain this interspace, and the PSI guide model was removed. Finally, a properly curved HTO plate was attached to the medial surface of the tibia as closely as possible, and the locking plate was tightly fixed by screws. Autogenous or allogenic bones were implanted if the lateral border of the osteotomy opening was larger than 10 mm (Figure 2).

As for conventional MOWHTO, under the guidance of intraoperative C-arm fluoroscope, the osteotomy sites were determined visually by the free hand of the senior surgeon (J.L.); the same two-plane osteotomy procedures were performed accordingly. The correction angle, hardware positioning, and accuracy were determined recurrently by the C-arm fluoroscope, and the exposures of radiography were recorded. The same criteria were applied for bone grafting.

### 2.4. Radiological and Arthroscopic Assessment

Radiological measurements were performed for both groups after surgery in the prementioned protocol (preoperatively, postoperatively, 3 months, and 12 months after surgery) by a single observer (YH.M.). All angles mentioned above were measured on the double-limb full-length standing position X-ray plain film (anteroposterior view), which is the benchmark of the measurement of the mechanical leg axis [23]. In the coronal plane, the mechanical femorotibial angle (mFTA, or weight-bearing line), the mechanical medial proximal tibial angle (mMPTA), and the mechanical lateral distal femoral angle (mLDFA) were measured. Correction errors for the mFTA and the mMPTA accounting for accuracy in the coronal plane were also calculated. Special attention was paid to correct the positioning of both legs/feet on the full-length standing X-ray views before angle measurements were undertaken. OA severity was scored according to the Kellgren–Lawrence scale. And upon the request of the internal fixation removal by patients, a concomitant arthroscopy was performed to assess the condition of intra-articular structures (cartilage, meniscus, ligaments, etc.)

### 2.5. Clinical and Functional Assessment

Commonly accepted patient-reported outcome measures including the International Knee Documentation Committee (IKDC) score and Lysholm score were used to assess the patients' subjective knee function. The subjective IKDC score is an 18-item, region-specific, patient-reported questionnaire containing the domains of symptoms, function, and sports activities [24]. The IKDC has been proven to be a valid and reliable instrument for patients who have knee injury and disability [25].

Intraoperative and postoperative adverse events up to 1 year were carefully documented for the assessment of technique safety. Common complications [8] including hinge fractures, delayed union/nonunion, infection, and deep vein thrombosis were strictly observed and duly managed. Visual analogue scale (VAS) was used to assess the preoperative pain and postoperative pain (24 hours, 48 hours, 1 month, 3 months, 6 months, and 12 months). The surgical duration for osteotomy, days of hospitalization, and dose of radiation (C-arm) were also recorded in every case. Standard follow-up with the senior surgeon (J.L.) was provided at 1 month, 3 months, 6 months, and 12 months postoperatively.

### 2.6. Statistical Analysis

All statistical tests were performed in Software Package for Social Sciences (SPSS) Statistics version 25.0. Categorical data were compared with Fisher exact tests. Continuous data were tested for normality and compared with either Student *t*-tests or Mann–Whitney tests depending on normality. A bivariate Spearman rank correlation was conducted to evaluate the relation between the mMPTA and mFTA in terms of effective correction. *P* values <0.05 were considered statistically significant.

## 3. Results

### 3.1. Radiological and Arthroscopic Outcomes

**Figure 3:**
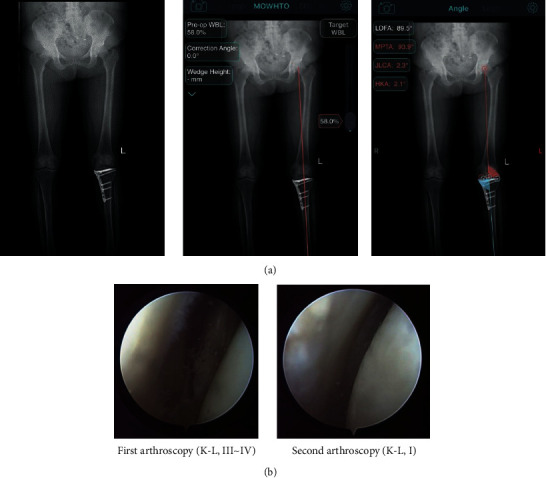
Full-length double-limb weight-bearing X-rays were taken for the assessment of the postoperative mFTA and mMPTA in the prementioned case, which were totally consistent with the target angles (a). The second arthroscopic look showed the cartilage degeneration recovered 18 months after surgery (b).

The postoperative full-length double-limb weight-bearing X-ray was regularly taken in all patients for the assessment of postoperative mFTA, mMPTA, and mLDFA. There were 3 patients in the 3D-printed PSI group and 4 patients in the conventional group requested for the removal of the internal fixation; all plates and screws were successfully removed, and concomitant arthroscopies were conducted. In 1 patient of the 3D-printed PSI group, arthroscopic results showed the cartilage degeneration recovered from the preoperative Outerbridge grade III to the postoperative Outerbridge grade I (Figure 3).

### 3.2. mFTA

**Figure 4:**
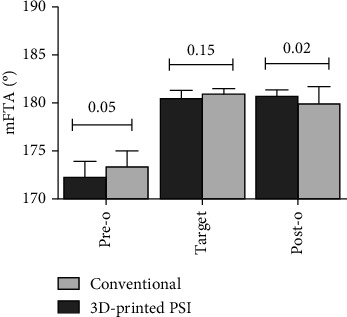
Preoperative, target, and postoperative mFTA measured at double-limb full-length standing position X-ray. mFTA: mechanical femorotibial angle; 3D: three-dimensional; PSI: patient-specific instrumentation; *P*_pre_ = 0.05; *P*_target_ = 0.15; *P*_post_ = 0.02.

The mFTA was corrected from a preoperative mean angle of 172.2° ± 1.7° to a postoperative mean angle of 180.7° ± 0.7° in the 3D-printed PSI group and from a preoperative mean angle of 173.3° ± 1.7° to a postoperative mean angle of 179.7° ± 1.8° in the conventional group. The PSI group preoperative planning for mFTA is to be corrected to 180.5° ± 0.91°. The postoperative results showed there was a larger absolute mFTA in the 3D group than the conventional group (*P* = 0.02). The mFTA correction in the 3D-printed PSI group was 8.5° ± 1.9°, which is significantly higher than the conventional group with a correction of 6.4° ± 1.90° (*P* = 0.0008) (Table 2). When compared to the target mFTA in the preoperational planning, the 3D-printed PSI group had a significantly smaller correction error than the conventional group (0.2 ± 0.6 vs. 1.2 ± 1.4, *P* = 0.004) (Figure 4).

### 3.3. mMPTA

**Figure 5:**
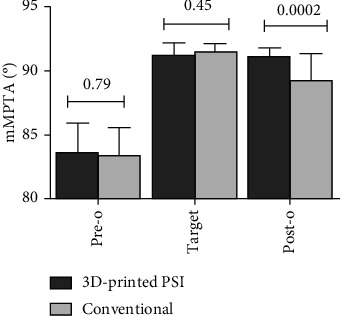
Preoperative, target, and postoperative mMPTA measured at double-limb full-length standing position X-ray. mMPTA: mechanical medial proximal tibial angle; 3D: three-dimensional; PSI: patient-specific instrumentation; *P*_pre_ = 0.79; *P*_target_ = 0.45; *P*_post_ = 0.0002.

The mMPTA was corrected from a preoperative mean angle of 86.3° ± 2.28° to a postoperative mean angle of 91.2° ± 0.65° in the 3D-printed PSI group and from a preoperative mean angle of 83.4° ± 2.15° to a postoperative mean angle of 89.3° ± 2.13° in the conventional group. The PSI group preoperative planning for mMPTA is to be corrected to 91.3° ± 0.87°. The postoperative results showed there was a larger absolute mMPTA in the 3D group than the conventional group (*P* = 0.0002). The mMPTA correction in the 3D-printed PSI group was 7.5° ± 2.16°, which is significantly higher than the conventional group with a correction of 5.9° ± 2.22° (*P* = 0.03). When compared with the preoperative target mMPTA, there was a significantly smaller correction error in the PSI group than in the conventional group (0.1 ± 0.4 vs. 2.2 ± 1.8, *P* < 0.00001) (Table 3) (Figure 5).

### 3.4. mLDFA

All patients in both groups did not meet the surgical indications for DFO. As for the preoperative and postoperative mLDFA in 3D-printed PSI group, the mean angles were 88.9° ± 1.86° and 89.0° ± 1.82°, respectively; there was no significant change observed in this group. No significant changes were observed in the conventional group in terms of preoperative and postoperative mLDFA; the mean angles were 89.4° ± 1.57 and 88.8° ± 1.85, respectively (Table 4).

### 3.5. Patient-Reported Outcomes and Clinical Outcomes

**Figure 6:**
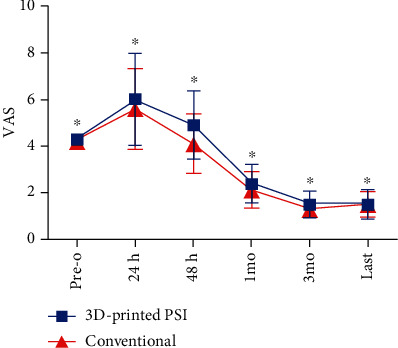
VAS at preoperative, 24 h, 48 h, 1 month, 3 months, and last follow-up after the operation. VAS: visual analogue scale (VAS; with 0, no pain, to 100, the worst imaginable pain); 3D: three-dimensional; PSI: patient-specific instrumentation; ^∗^*P* > 0.05; Pre-o: preoperative.

In every case, a successful surgical procedure was conducted, and no intraoperative complications were observed, while the exposures of intraoperative C-arm fluoroscopy in the PSI group (1.3 ± 0.12) were significantly smaller than the conventional group (4.1 ± 0.57) (*P* < 0.00001). Moreover, there was a significantly shorter time for the osteotomy procedure in the PSI group (37.8 ± 7.14) than in the conventional group (54.6 ± 11.72) (*P* < 0.00001), and this allowed more concomitant treatments. No significant differences were found in the VAS scores postoperatively at each time point (Figure 6); neither was found in hospitalization days. There were 2 patients in the conventional group caught up with lateral hinge fracture at the 1-month follow-up, delayed weight-bearing and moderate rehabilitation protocols were made for them. There were 3 patients in the conventional group and one patient in the PSI group detected to have intermuscular venous thrombosis by ultrasound postoperatively (color Doppler ultrasound examinations of the lower extremity were performed 3 days after surgery regularly); no special anticoagulant therapy was applied, and those patients were asymptomatic at each follow-up. Minor local infection signs were found in one PSI patient at the osteotomy site, which was probably caused by allogenic bone graft; the infection was controlled by antibiotics and immobilization. One patient in the conventional group had a postoperative intra-articular infection, debridement under arthroscopy was conducted, adequate drainage and antibiotic therapy were also applied, and the patient fully recovered afterwards (Table 5).

**Figure 7:**
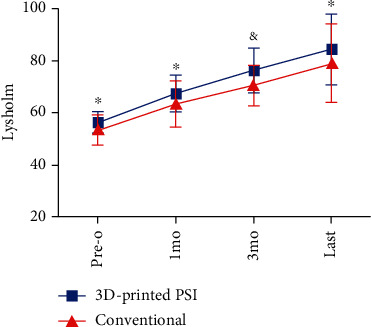
Preoperative and postoperative Lysholm scores at 1 month, 3 months, and last follow-up. 3D: three-dimensional; PSI: patient-specific instrumentation; pre-o: preoperative; mo: month. ^∗^*P*_pre−o,1mo,last_ > 0.05. ^&^*P*_3mo_ = 0.03.

**Figure 8:**
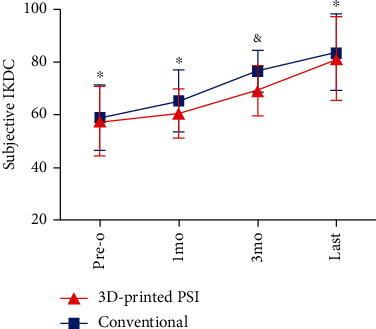
Preoperative and postoperative subjective IKDC scores at 1 month, 3 months, and last follow-up. 3D: three-dimensional; PSI: patient-specific instrumentation; pre-o: preoperative; mo: month. ^∗^*P*_pre−o,1mo,last_ > 0.05. ^&^*P*_3mo_ = 0.009.

As for patient-reported functional measurements, there were significantly higher scores observed in the 3D-printed PSI group than the conventional group in terms of both subjective IKDC score (76.6 ± 7.9 vs. 69.1 ± 9.6, *P* = 0.009) and Lysholm score (76.4 ± 8.9 vs. 70.4 ± 7.8, *P* = 0.03) at the 3-month follow-up. No significant differences regarding both the IKDC scores and Lysholm scores were noticed between the two groups at other times of follow-up (Figures 7 and 8).

## 4. Discussion

The goal of MOWHTO is to change the abnormal load of the medial knee compartment in patients with varus deformity and prevent the further development of osteoarthritis [26–28]. By correcting the alignment, MOWHTO evenly distributed the excessive load from the lower medial compartment to the whole articular surface [12, 28]. The general aim was to bring the weight-bearing axis to 62.5% of the proximal tibia width [29], but more recent biomechanical and clinical studies advocate a less aggressive overcorrection [6, 30, 31]. In this study, a 55%~60% proximal tibial width as the target weight-bearing axis was chosen. On the purpose of preserving a native knee joint, MOWHTO is an effective procedure of postponing the requirement of partial or total knee arthroplasty [7, 32] and creates the probability of cartilage recovery. The precision of the osteotomy is one of the cornerstones for successful OWHTO surgery. Conventional HTO planning and execution is commonly performed on two-dimensional radiographs [33] (X-rays, C-arm), and in face of deformities on both sagittal and coronal planes, the traditional technique seems to be incompetent and prone to error [34]. Moreover, the hip-knee-ankle angle (HKA), which is used to plan HTO, was reported to be inconsistent preoperatively, intraoperatively, and postoperatively in most cases [35, 36]; this is due to the variation in both knee rotation and flexion under different circumstances. In the era of precision medicine, the lack of consistency in conventional MOWHTO is probably the biggest barrier for this technique to become widely accepted [17].

The most important finding of this study is that this novel 3D-printed PSI technique is capable of delivering a higher level of accuracy in angular correction than conventional techniques. By the hand of an experienced surgeon, though the postoperative mFTAs of the conventional HTO also achieved the “acceptable range” (valgus from 3° to 6°) mentioned by Hernigou et al. [37]; nevertheless, there was a significantly shorter operation duration in the PSI group than the conventional technique. In addition to the improvement of accuracy and surgical duration, the PSI technique is a safer approach with higher feasibility for fewer complications and adverse events occurred in the 3D-printed PSI group, and there was a lower dosage of radiation brought by intraoperative C-arm scanning. These merits not only allow more concomitant treatment procedures (debridement, meniscectomy, ACLR, etc.) but also ensure enhanced recovery after surgery. To our knowledge, only a few studies have been reporting feasibility and accuracy outcomes about the clinical use of PSI in osteotomy around the knee [5, 15, 17, 19, 20, 22]. In the study by Van Genechten et al. [5], the two planar MOWHTOs were performed in a relatively conventional manner (freehand), while a PSI 3D-printed wedge and cast were adopted instead of the HTO plate. Interestingly, they also got excellent corrections outcomes; this precision was achieved by the patient-specific wedge model fixation rather than the osteotomy procedure itself. As such, the accuracy of the precised MOWHTO can be achieved in more than one way with the assistance of the PSI 3D-printed technique. In earlier laboratory studies, the finite element analysis (FEA) model by Chieh-Szu et al. [21] indicated there was a significant reduction of compressive load on the tibial plateau in their PSI osteotomy knees when compared with conventional ones (78.8 MPa vs. 91.9 MPa, under 600-N force); it revealed the PSI technique was capable of improving the structural stability, and this novel approach may have the potential to reduce the incidence of hardware dislocation and hinge fractures. In all, although the techniques of PSI and execution of related HTOs varied greatly, the outcomes turned favourable for PSI 3D-printed technique in all existing studies. However, the accuracy and clinical advantage of PSI over the conventional surgical methodology in MOWHTO still needs to be proven in large comparative studies with long-term follow-up.

Moreover, the effective treatment for knee OA is not merely about the correction of malalignment; further attention should be paid to the intra-articular illness. A visual assessment under arthroscopy can provide a more effective diagnosis of cartilage degeneration. In addition, treatment for the concomitant disease of OA (such as loose body, synovitis, meniscus injury, and ACLR) can also be practiced arthroscopically. A comprehensive surgical treatment merits further focus; we should not be limited to isolated osteotomy. Besides, to obtain robust immediate postoperative stability and biomechanics, autogenous bone grafting was recommended in cases with the wedge opening higher than 10 mm, and a crossing screw may also be considered; thus, enhanced recovery after surgery can be achieved.

## 5. Conclusion

With the assistance of 3D-printed PSI, a safe and feasible MOWHTO can be conducted with superior accuracy than the conventional techniques. The combination of precise 3D osteotomy cutting guide model contributed to a more accurate translation from planning to surgery, and a shorter operation duration created the opportunities for more concomitant treatments.

## Figures and Tables

**Table 1:** Demographic characteristics of the two groups.

	3D-printed PSI (mean ± SD)	Conventional (mean ± SD)	*P* value
Age, years	44.2 ± 11.7	41.8 ± 10.2	n.s
Sex (male : female)	4 : 14	5 : 14	n.s
Right : left	11 : 7	13 : 6	n.s
BMI	25.6 ± 3.68	25.1 ± 3.91	n.s
ROM (°)	126 ± 11.2	121 ± 10.3	n.s
mFTA (°)	172.2 ± 1.7	172.0 ± 1.9	n.s
mMPTA (°)	86.3° ± 2.28°	83.4° ± 2.15°	n.s
mLDFA (°)	88.9 ± 1.86	89.4 ± 1.57	n.s
OA Kellgren–Lawrence grading (I : II : III)	2 : 8 : 8	4 : 5 : 10	—
Planned wedge opening (mm)	8.9 ± 1.1	8.5 ± 1.5	n.s
Meniscus injury (*n*)	7	8	n.s
ACL injury (*n*)	2	3	n.s

Abbreviations: 3D: three-dimensional; PSI: patient-specific instrumentation; SD: standard deviation; n.s: not significant; BMI: body mass index; ROM: range of motion; mFTA: mechanical femorotibial angle; mMPTA: mechanical medial proximal tibial angle; mLDFA: mechanical lateral distal femoral angle; OA: osteoarthritis; ACL: anterior cruciate ligament.

**Table 2:** Preoperative, target, and postoperative mFTA measured at double-limb full-length standing position X-ray.

	3D-printed PSI (*n* = 18)	Conventional (*n* = 19)	*P* value
mFTA (°)			
Correction angle	8.5 ± 1.9	6.4 ± 1.9	*P* = 0.0008
Correction error	0.2 ± 0.6	1.2 ± 1.4	*P* = 0.004

mFTA: mechanical femorotibial angle; 3D: three-dimensional; PSI: patient-specific instrumentation; *P*_pre_ = 0.05; *P*_arget_ = 0.15; *P*_post_ = 0.02.

**Table 3:** Preoperative, target, and postoperative mMPTA measured at double-limb full-length standing position X-ray.

	3D-printed PSI (*n* = 18)	Conventional (*n* = 19)	*P* value
mMPTA (°)			
Correction angle	7.5 ± 2.2	5.9 ± 2.2	*P* = 0.03
Correction error	0.1 ± 0.4	2.2 ± 1.8	*P* < 0.00001

mMPTA: mechanical medial proximal tibial angle; 3D: three-dimensional; PSI: patient-specific instrumentation; *P*_pre_ = 0.79; *P*_target_ = 0.45; *P*_post_ = 0.0002.

**Table 4:** mLDFA.

	3D-printed PSI (*n* = 18)	Conventional (*n* = 19)
mLDFA (°)		
Preoperative	88.9 ± 1.86	89.4 ± 1.57
Postoperative	89.0 ± 1.82	88.8 ± 1.85
*P* value	n.s	n.s

Abbreviations: mFTA: mechanical femorotibial angle; mMPTA: medial mechanical proximal tibial angle; mLDFA: mechanical lateral distal femoral angle; n.s: not significant; 3D: three-dimensional; PSI: patient-specific instrumentation.

**Table 5:** Clinical outcomes.

	3D-printed PSI	Conventional	*P* value
Feasibility			
Operation time of osteotomy (min)	37.8 ± 7.14	54.6 ± 11.72	*P* < 0.00001
Radiation exposures (*n*)	1.3 ± 0.12	4.1 ± 0.57	*P* < 0.00001
Hospitalization (d)	5.6 ± 1.28	6.2 ± 1.34	n.s
Bone graft	2.1 ± 0.33	2.2 ± 0.37	n.s

Complications (*n*)			
Displaced (>2 mm) lateral hinge fracture	0	0	—
Undisplaced (<2 mm) lateral hinge fracture	0	2	—
Deep vein thrombosis	1	3	—
Infection	1	1	—
Hardware failure	0	0	—

Abbreviations: VAS: visual analogue score; n.s: not significant; 3D: three-dimensional; PSI: patient-specific instrumentation.

## Data Availability

The results in this study are available from the corresponding author on reasonable request.

## References

[1] Darees M., Putman S., Brosset T., Roumazeille T., Pasquier G., Migaud H. (2018). Opening-wedge high tibial osteotomy performed with locking plate fixation (TomoFix) and early weight-bearing but without filling the defect. A concise follow-up note of 48 cases at 10 years' follow-up. *Orthopaedics & Traumatology, Surgery & Research*.

[2] Hantes M. E., Natsaridis P., Koutalos A. A., Ono Y., Doxariotis N., Malizos K. N. (2018). Satisfactory functional and radiological outcomes can be expected in young patients under 45 years old after open wedge high tibial osteotomy in a long-term follow-up. *Knee Surgery, Sports Traumatology, Arthroscopy*.

[3] Bannuru R. R., Osani M. C., Vaysbrot E. E. (2019). OARSI guidelines for the non-surgical management of knee, hip, and polyarticular osteoarthritis. *Osteoarthritis and Cartilage*.

[4] Van den Bempt M., Van Genechten W., Claes T., Claes S. (2016). How accurately does high tibial osteotomy correct the mechanical axis of an arthritic varus knee? A systematic review. *The Knee*.

[5] van Genechten W., van Tilborg W., Van den Bempt M., Van Haver A., Verdonk P. (2020). Feasibility and 3D Planning of a Novel Patient-Specific Instrumentation Technique in Medial Opening-Wedge High Tibial Osteotomy. *The Journal of Knee Surgery*.

[6] Martay J. L., Palmer A. J., Bangerter N. K. (2018). A preliminary modeling investigation into the safe correction zone for high tibial osteotomy. *The Knee*.

[7] Konopka J. F., Gomoll A. H., Thornhill T. S., Katz J. N., Losina E. (2015). The cost-effectiveness of surgical treatment of medial unicompartmental knee osteoarthritis in younger patients. *The Journal of Bone and Joint Surgery. American Volume*.

[8] Martin R., Birmingham T. B., Willits K., Litchfield R., Lebel M. E., Giffin J. R. (2014). Adverse event rates and classifications in medial opening wedge high tibial osteotomy. *The American Journal of Sports Medicine*.

[9] Elson D. W. (2017). The surgical accuracy of knee osteotomy. *The Knee*.

[10] Wu Z. P., Zhang P., Bai J. Z. (2018). Comparison of navigated and conventional high tibial osteotomy for the treatment of osteoarthritic knees with varus deformity: a meta-analysis. *International Journal of Surgery*.

[11] Han S. B., Lee D. H., Shetty G. M., Chae D. J., Song J. G., Nha K. W. (2013). A "safe zone" in medial open-wedge high tibia osteotomy to prevent lateral cortex fracture. *Knee Surg Sports Traumatol Arthrosc*.

[12] Sprenger T. R., Doerzbacher J. F. (2003). Tibial osteotomy for the treatment of varus gonarthrosis. Survival and failure analysis to twenty-two years. *The Journal of Bone and Joint Surgery. American Volume*.

[13] Jones G. G., Jaere M., Clarke S., Cobb J. (2018). 3D printing and high tibial osteotomy. *EFORT Open Rev*.

[14] Sarment D. P., Al-Shammari K., Kazor C. E. (2003). Stereolithographic surgical templates for placement of dental implants in complex cases. *The International Journal of Periodontics & Restorative Dentistry*.

[15] Kim H. J., Park J., Shin J. Y., Park I. H., Park K. H., Kyung H. S. (2018). More accurate correction can be obtained using a three-dimensional printed model in open-wedge high tibial osteotomy. *Knee Surgery, Sports Traumatology, Arthroscopy*.

[16] Lu S., Zhang Y. Z., Wang Z. (2012). Accuracy and efficacy of thoracic pedicle screws in scoliosis with patient-specific drill template. *Medical & Biological Engineering & Computing*.

[17] Victor J., Premanathan A. (2013). Virtual 3D planning and patient specific surgical guides for osteotomies around the knee. *Bone Joint J*.

[18] Donnez M., Ollivier M., Munier M. (2018). Are three-dimensional patient-specific cutting guides for open wedge high tibial osteotomy accurate? An in vitro study. *Journal of Orthopaedic Surgery and Research*.

[19] Munier M., Donnez M., Ollivier M. (2017). Can three-dimensional patient-specific cutting guides be used to achieve optimal correction for high tibial osteotomy? Pilot study. *Orthopaedics & Traumatology, Surgery & Research*.

[20] Pérez-Mañanes R., Burró J., Manaute J., Rodriguez F., Martín J. (2016). 3D Surgical Printing Cutting Guides for Open-Wedge High Tibial Osteotomy: Do It Yourself. *The Journal of Knee Surgery*.

[21] Chieh-Szu Yang J., Chen C. F., Lee O. K. (2020). Benefits of opposite screw insertion technique in medial open-wedge high tibial osteotomy: a virtual biomechanical study. *J Orthop Translat*.

[22] Yang J. C.-S., Chen C.-F., Luo C.-A. (2018). Clinical Experience Using a 3D-Printed Patient-Specific Instrument for Medial Opening Wedge High Tibial Osteotomy. *BioMed Research International*.

[23] Sharma L., Song J., Felson D. T., Cahue S., Shamiyeh E., Dunlop D. D. (2001). The role of knee alignment in disease progression and functional decline in knee osteoarthritis. *JAMA*.

[24] Irrgang J. J., Anderson A. F., Boland A. L. (2017). Development and Validation of the International Knee Documentation Committee Subjective Knee Form. *The American Journal of Sports Medicine*.

[25] Higgins L. D., Taylor M. K., Park D. (2007). Reliability and validity of the international knee documentation committee (IKDC) subjective knee form. *Joint, Bone, Spine*.

[26] Bauer G., Insall J., Koshino T. (1969). Tibial osteotomy in gonarthrosis (osteo-arthritis of the knee). *The Journal of Bone and Joint Surgery. American Volume*.

[27] Insall J. N., Joseph D. M., Msika C. (1984). High tibial osteotomy for varus gonarthrosis. A long-term follow-up study. *The Journal of Bone and Joint Surgery. American Volume*.

[28] Yasuda K., Majima T., Tsuchida T., Kaneda K. (1992). A ten- to 15-year follow-up observation of high tibial osteotomy in medial compartment osteoarthrosis. *Clin Orthop Relat Res*.

[29] DUGDALE T. H. O. M. A. S. W., NOYES F. R. A. N. K. R., STYER D. A. V. I. D. (1992). Preoperative Planning for High Tibial osteotomy. The effect of lateral tibiofemoral separation and tibiofemoral length. *Clinical Orthopaedics and Related Research*.

[30] Stanley J. C., Robinson K. G., Devitt B. M. (2016). Computer assisted alignment of opening wedge high tibial osteotomy provides limited improvement of radiographic outcomes compared to flouroscopic alignment. *The Knee*.

[31] van de Pol G. J., Verdonschot N., van Kampen A. (2012). The value of the intra-operative clinical mechanical axis measurement in open-wedge valgus high tibial osteotomies. *The Knee*.

[32] Smith W. B., Steinberg J., Scholtes S., Mcnamara I. R. (2017). Medial compartment knee osteoarthritis: age-stratified cost-effectiveness of total knee arthroplasty, unicompartmental knee arthroplasty, and high tibial osteotomy. *Knee Surgery, Sports Traumatology, Arthroscopy*.

[33] Brinkman J., Lobenhoffer P., Agneskirchner J., Staubli A., Wymenga A., van Heerwaarden R. (2008). Osteotomies around the knee: patient selection, stability of fixation and bone healing in high tibial osteotomies. *The Journal of Bone and Joint Surgery. British Volume*.

[34] Kawakami H., Sugano N., Yonenobu K. (2004). Effects of rotation on measurement of lower limb alignment for knee osteotomy. *Journal of Orthopaedic Research*.

[35] Koshino T., Takeyama M., Jiang L. S., Yoshida T., Saito T. (2002). Underestimation of varus angulation in knees with flexion deformity. *The Knee*.

[36] Swanson K. E., Stocks G. W., Warren P. D., Hazel M. R., Janssen H. F. (2000). Does axial limb rotation affect the alignment measurements in deformed limbs?. *Clin Orthop Relat Res*.

[37] Hernigou P., Medevielle D., Debeyre J., Goutallier D. (1987). Proximal tibial osteotomy for osteoarthritis with varus deformity. A ten to thirteen-year follow-up study. *The Journal of Bone and Joint Surgery. American Volume*.

